# Collective self-assessment in banded mongoose intergroup contests

**DOI:** 10.1093/beheco/araf133

**Published:** 2025-11-21

**Authors:** C W Rayner, P A Green, K L Hunt, F J Thompson, F Mwanguhya, M A Cant, D W E Sankey

**Affiliations:** Centre for Ecology and Conservation, Faculty of Environment, Science and Economy, University of Exeter, Penryn Campus, Cornwall TR10 9FE, United Kingdom; Department of Ecology, Evolution, and Organismal Biology, Brown University, Providence, RI 02912, United States; Centre for Ecology and Conservation, Faculty of Environment, Science and Economy, University of Exeter, Penryn Campus, Cornwall TR10 9FE, United Kingdom; Centre for Ecology and Conservation, Faculty of Environment, Science and Economy, University of Exeter, Penryn Campus, Cornwall TR10 9FE, United Kingdom; Banded Mongoose Research Project, Queen Elizabeth National Park, Kasese District, Rubirizi, Uganda; Centre for Ecology and Conservation, Faculty of Environment, Science and Economy, University of Exeter, Penryn Campus, Cornwall TR10 9FE, United Kingdom; Centre for Ecology and Conservation, Faculty of Environment, Science and Economy, University of Exeter, Penryn Campus, Cornwall TR10 9FE, United Kingdom; School of Natural and Environmental Science, Newcastle University, Newcastle upon Tyne NE1 7RU, United Kingdom

**Keywords:** animal contests, banded mongoose, contest theory, intergroup conflict, self-assessment

## Abstract

Contests over resources are widespread in nature. To optimize outcomes, animals assess fighting abilities, deciding to escalate conflicts based on their own strength (self-assessment) or comparing their own strength with that of their rival (mutual assessment). While most research focuses on one-on-one (dyadic) contests, the assessment strategies employed by groups remain poorly understood, even though animal groups from ants to humans engage in intergroup conflict. Mutual assessment is frequently assumed, as more information is thought to improve decision-making; however, this assumption has rarely been tested. Here we used a dataset spanning 21 years and 633 intergroup contests in a banded mongoose (*Mungos mungo*) population in Queen Elizabeth National Park, Uganda. Our results support a model of self-assessment: groups with many males tend to escalate conflicts regardless of the rival group's strength, thus contrasting the commonly held assumption that decisions during intergroup contests are made by mutual assessment. We suggest that assessing rival group strength during conflict could be disproportionately costly, compared with assessing own group strength, which can be done over longer time periods and is easier to obtain. Greater understanding of these dynamics can shed light on the drivers and escalation patterns of intergroup conflict across social species, including humans.

## Teaser

When two rival groups come together, what determines how they fight? We found support for the hypothesis that banded mongoose groups escalate into physical fights based upon an estimation of their own group's strength, which we term collective self-assessment. We did not find evidence for a commonly held notion that groups compare their own group's strength with their rival's. Distinguishing between assessment strategies is intensively researched in contests between individuals but has rarely been applied to intergroup conflict. Here we applied the successful “assessment strategy framework” to group conflict, analyzing over 20 years of data from over 600 intergroup interactions. We suggest that collective self-assessment is employed because it is faster than mutual assessment, which offers an advantage in conflicts where time is of the essence.

## Introduction

Natural resources can be limited and unevenly distributed both in time and space. This creates competition for those resources when they can be monopolized (Nagel 1995; Grover 1997; Wrangham 1999; Bornstein 2003; De Dreu et al. 2020; Rusch and Gavrilets 2020; De Dreu and Triki 2022; Mullon and Lehmann 2022). Contests are one way to settle disputes over such resources and are widespread across taxa (Briffa et al. 2013). Although the benefits from winning contests can lead to increases in fitness (Le Boeuf 1974; De Dreu and Triki 2022), the costs of escalation can be as impactful; for example, through the loss of energy (Payne and Pagel 1996, 1997; Hack 1997; Briffa and Sneddon 2007), and risk of injury (Payne 1998; Lane and Briffa 2017) or death (Enquist and Leimar 1990; Wrangham et al. 2006; Harbom et al. 2008; Thompson et al. 2017). As such, evolutionary theory predicts that contestants should minimize the costs and maximize the benefits when deciding whether to fight, and to what degree of escalation (Parker and Smith 1990; Birch 2016; Budaev et al. 2019).

To respond optimally and reduce uncertainty animals can assess fighting ability (also termed resource holding potential (RHP)) (Parker 1974; Smith and Parker 1976; Parker and Rubenstein 1981; Arnott and Elwood 2008, 2009). Studies of assessment in dyadic contests have shown that individuals can assess many different RHP-contributing features of themselves and their opponents (eg, (Morrell et al. 2005; Arnott and Elwood 2009; Green and Patek 2018; Pinto et al. 2019; Green et al. 2021)). This can include morphology, such as assessments of snout-to-vent length in male yellow-headed geckos (*Gonatodes albogularis*) (Bohórquez-Alonso et al. 2014), or body size as seen in models of contest theory (Palaoro and Briffa 2017). Physiology may also be assessed through metabolic costs (Briffa and Sneddon 2007), which has been suggested in male convict cichlids (*Amatitlania nigrofasciata*) (Copeland et al. 2011). Behaviors, such as aggressiveness, may also be assessed by animals such as in pigs (*Sus scrofa*) (Camerlink et al. 2015) and green swordtails (*Xiphophorus hellerii*) (Wilson et al. 2011). This is well studied in contests between individuals (ie, dyadic contests), but much less is known about assessment in contests between groups (but see (Briffa 2014).

**Fig. 1.**
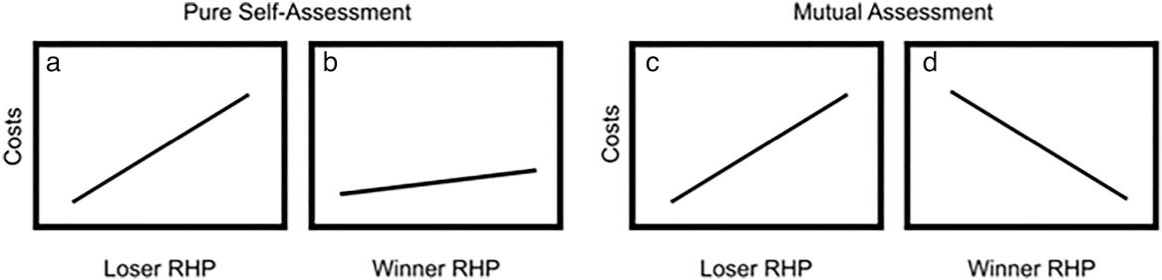
Assessment strategy framework (Arnott and Elwood 2009). Pure self-assessment predicts (a) a positive relationship between loser RHP and costs (eg, duration, likelihood of escalation), and (b) a weaker positive relationship between winner RHP and costs. Mutual assessment predicts (c) a positive relationship between loser RHP and costs, and (d) a negative relationship between winner RHP and costs.

When groups gather information to make conflict decisions, do they assess own strength (self-assessment) or do they assess both their own strength and the strength of the other group (mutual assessment)? In dyadic contests, the “assessment strategy framework” has been a widely utilized framework to differentiate between strategies (ie, self and mutual assessment) used to assess RHP ((Arnott and Elwood 2009); Fig. 1). It has been suggested that this framework can be fruitfully extended to intergroup contests (Green et al. 2021). Yet, most intergroup contest studies do not test or evaluate alternative assessment models. Some experimental studies that use presentations (playbacks or scent marks) have been fundamental in probing information-gathering during intergroup contests, but as no actual contest occurs during presentations it is not possible to relate them directly to how this information-gathering influences the decisions made in contests (Müller and Manser 2007; Herbinger et al. 2009; Benson-Amram et al. 2011; Furrer et al. 2011; Spezie et al. 2023). To the best of our knowledge the assessment strategy framework has not been directly tested in any social species outside of humans (Briffa 2014). Adapting the assessment strategy framework (Arnott and Elwood 2009) to intergroup contests can reveal potentially shared principles across levels of biological organization (individuals to groups), while suggesting how groups come to collaborative decisions during contests.

The assessment strategy framework makes assumptions and predictions about the drivers of contest behaviors, many of which can be tested by comparing relationships between competitor RHP and contest costs (eg, duration, likelihood of escalation; summarized in Fig. 1). If animals use a pure self-assessment strategy, contests will be won by those with the greater cost threshold, determined by their RHP, and no information about the opponent is assessed (Maynard Smith 1974; Green et al. 2021). Contest costs are driven mostly by the fighting ability of losers, since winners only need to withstand a higher cost than their opponent (Fig. 1a, b) (Mesterton-Gibbons et al. 1996; Payne and Pagel 1996, 1997). In mutual assessment, a loser forfeits not when costs reach a certain limit, but as they discern that they have a lower RHP than their rival (Enquist and Leimar 1983, 1987; Enquist et al. 1990). As in self-assessment, high RHP losers can incur higher costs (Fig. 1c), yet, as opponents are assessed, high RHP winners are more quickly identified by the loser (Fig. 1d). It is worth noting that some species can fit more than one model, depending on context, that individuals can change strategies during a contest (Lobregat et al. 2019; Dinh et al. 2020; Chen et al. 2022), and that individuals within a population may vary in their assessment strategy (Chapin et al. 2019).

When applying the assessment strategy framework to the group context, we assume that group members each choose the conflict costs they are willing to incur based only on an assessment of their own group's RHP (collective self-assessment), or the RHP of their own and the rival group's RHP (collective mutual assessment), and that these individual choices combine to determine the decision of the group. Note as a shorthand we describe groups as taking decisions or using assessment strategies, without assuming that groups are agents in themselves (Okasha 2018).

**Fig. 2.**
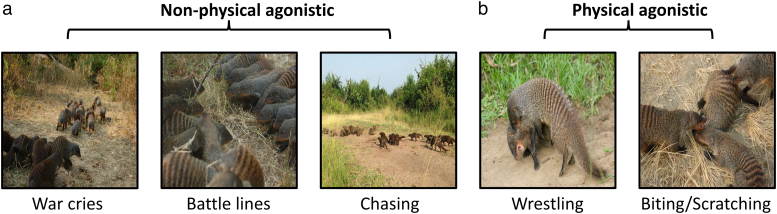
Categories of agonistic behavior observed during intergroup contests in banded mongooses. (a) Agonistic behaviors such as war crying, forming battle lines, and chasing were considered as a “non-physical” level of intensity; (b) Agonistic behaviors such as biting, scratching, wrestling, or where aggressive physical contact was observed was considered as a “physical” level of intensity. Injurious and lethal violence, where aggressive physical contact resulted in severe or fatal injury or death was also included as a “physical” level of intensity. (Image credit from left to right: Harry Marshall, Harry Marshall, Harry Marshall, Dave Seager, and Harry Marshall).

Here, we use the assessment strategy framework to test which assessment strategy is used in escalation during intergroup contests in banded mongooses (*Mungos mungo*)—a cooperatively breeding species which hold territories and fight fiercely over resources (Cant et al. 2013, 2016) such as estrus females (Green et al. 2025) and food (Thompson et al. 2017). Banded mongooses are an ideal species to test for intergroup contest assessment strategy for several reasons. Firstly, banded mongoose intergroup contests have significant fitness consequences. Intergroup conflict in this species is responsible for 10% or more of adult deaths and 20% of infant deaths with identifiable causes, which in mammals is matched only by chimpanzees, wolves, lions, and some human societies (Wrangham et al. 2006; Cubaynes et al. 2014; Johnstone et al. 2020). These high stakes likely impose strong selection pressures on assessment strategies. Second, contests have a range of escalating intensities from non-physical (eg, war crying, forming battle lines, chasing) to physical (eg, biting, scratching, wrestling) including injurious lethal violence (Cant et al. 2016) (Fig. 2). This variance in intensity (a proxy for cost) is essential for testing assessment strategy (Arnott and Elwood 2009), and similar metrics of escalation have been used in studies of dyadic assessment strategies (Yasuda et al. 2012; McGinley et al. 2015; Green and Patek 2018). Finally, we have known proxies for banded mongoose group RHP, which is a crucial prerequisite for testing assessment of RHP. We know that contest success is most strongly determined by the number of adult males in the group and age of the group's oldest male (Green et al. 2022). Overall, the intensity and variability of conflict, and our solid baseline understanding of RHP proxies set the stage for banded mongooses to be an ideal model for testing assessment strategies in intergroup contests.

In this study we tested collective self-assessment and collective mutual assessment as two alternative hypotheses that describe patterns of escalation in banded mongoose contests. Following common assumptions in intergroup conflict (ie, animals compare each other's fighting abilities), we predicted that groups would use mutual assessment. Although more cognitively demanding, the use of more information from mutual assessments could allow groups to give up earlier when clearly outmatched in fights and is often assumed the superior or default strategy for intergroup assessment (Bennett and Stam 1996; Chan 2003; Radford 2003; Langlois and Langlois 2009; Elwood and Arnott 2012; Stulp et al. 2012; Van Belle and Scarry 2015). For our measure of costs, we used the degree of escalation, from non-physical to physical (Fig. 2). If banded mongooses used collective self-assessment, we predict the previously identified RHP proxies—(i) number of adult males or (ii) the age of the oldest male (Green et al. 2022)—would be significantly positively correlated with the probability of escalation to physical violence for losing groups and would show a weaker positive relationship in winning groups (Fig. 1a, b). Conversely, if, as predicted, banded mongooses do employ collective mutual assessment, we expect these same variables to exhibit a significant positive correlation in losing groups and a significant negative correlation in winning groups (Fig. 1c, d).

## Methods

### Study population and data collection

All data was collected as part of the Banded Mongoose Research Project, a long-term study on a population of banded mongooses in and around Mweya Peninsula, Queen Elizabeth National Park, Uganda (0°12′S, 29°54′E). This study includes data collected from 19th February 2000 to 1st April 2021 encompassing 633 intergroup contests from 39 different groups and 76 unique pairings of groups. In general, at any given time, there are approximately 250 individuals present within the population making up 10 to 12 groups consisting of around 10 to 30 adults each (Cant 2000; Cant et al. 2013). Every 1 to 3 days researchers visited groups to record data on life-history (eg, births, deaths), composition of each group, and details about their intergroup interactions (below).

### Scoring intergroup contest intensity and escalation

Intergroup contests were recorded opportunistically as they occurred. Intergroup contests were defined as any time when at least two groups directed agonistic behavior toward each other (Fig. 2). Data on contest duration was not available. Intensity and escalation of intergroup contests were used instead as a proxy for cost and scored using comments recorded by the Banded Mongoose Research Project team. As an intergroup contest often involves a series of behaviors in which intensity escalates until one or both groups retreat (at which point the intergroup contest ends) the highest point of escalation was used as the level of intensity for each contest. The group that retreated was defined as the loser group. For ease of analysis, the range of intensities possible during an intergroup contest was divided into two categories (Fig. 2). The lowest level of intensity was “non-physical” which was defined as an instance where two groups directed non-physical agonistic behavior (eg, vocal and visual displays; (Cant et al. 2016)) toward each other and/or fled upon sighting. If this then escalated to fighting between the two groups in which there was aggressive physical contact (eg, biting, scratching, wrestling; (Cant et al. 2016)), this was defined as “physical”. Intergroup contests which escalate to physical combat are expected to have higher contest costs (such as energy used and injury risk) compared to non-physical contests (McGinley et al. 2015; Lane and Briffa 2017; Green and Patek 2018). Because the original dataset did not include enough information on whether only one group attempted to escalate, escalation was assumed to reflect a decision (or at least participation) by both groups, regardless of which group initiated the interaction. This approach follows the logic that even losing groups must have engaged physically to some degree and therefore incurred the associated energetic and injury risks. A more detailed behavioral analysis (eg, whether only a subset of individuals from each group participated at any stage) was unavailable, but notably some dyadic contest studies have used overall metrics of escalation to test assessment in a similar fashion (eg, (Green and Patek 2018)). Our scoring approach was then translated into a binomial escalation metric with 0 representing non-physical and 1 representing physical. Injurious and lethal violence in which aggressive physical contact results in severe injury or death of one or more individuals was included in the “physical” category, rather than a category of its own. This is because, firstly, injury and mortality were data poor (9.4% of all intergroup interactions; *N* = 60), but more importantly, whether individuals suffer an injury is not so much a decision as it is a (potentially random) outcome of such physical combat. By contrast, whether violence escalates from non-physical to physical combat is a decision the group may make. Therefore, our use of the escalation metric likely reflects the cost each group was willing to pay.

For each intergroup contest, a qualitative comment (a description of the events of the contest) was recorded by observers in the field. These comments were then assessed by three researchers (CR, FM, DS) to evaluate whether the contest escalated into physical violence (with 0 representing non-physical and 1 representing physical), or whether the comments did not allow us to determine whether or not the contest escalated (termed: “undeterminable”). Second, all researchers assigned a confidence score (1 to 3) to each of their categorizations of intensity score, reflecting their certainty in the intensity score. The confidence score was based on criteria such as the clarity and detail of the recorded comment, as well as contextual factors that might influence interpretation (eg, visibility conditions or proximity to the event). A score of 1 indicated high confidence, 2 indicated moderate confidence, and 3 indicated low confidence. We ended up removing contests that scored with low confidence (3) from the dataset because they were missing data on other variables included as fixed effects in our analysis. The three researchers discussed any ambiguous comments in detail to assign an escalation and confidence score if possible. The discussion was guided by FM, who has over 27 years of experience observing and collecting field data on banded mongooses and managing the Banded Mongoose Research Project.

We report models using both highly confident and moderately confident scores (1,2), and models including only highly confident scores (1).

### Statistical analysis

All statistical analyses were carried out using *R* 4.01 (R Core Team 2021). Binomial escalation response data were analyzed using generalized linear mixed effect models (GLMMs) with a binomial error structure and a logit link function (Bolker et al. 2009) with the “lme4' package (Bates et al. 2015). For both losing groups and winning groups, we tested the effect of two RHP predictor variables—number of adult males (>6 months old) and age of the oldest male (days) (Green et al. 2022). Both variables were scaled with mean of zero and unit variance using the scale function (Becker et al. 1988).

In total, we ran two GLMM models, one model using data from high and moderate confidence scores, and another using data from only high confidence scores. The model for each confidence category is as follows (“∼” represents “as predicted by”):

$$\begin{array}{ll} \mathrm{escalation}∼ & \mathrm{loser}\;\mathrm{number}\;\mathrm{of}\;\mathrm{adult}\;\mathrm{males}\\ & +\mathrm{loser}\;\mathrm{age}\;\mathrm{of}\;\mathrm{oldest}\;\mathrm{male}\\ & +\mathrm{winner}\;\mathrm{number}\;\mathrm{of}\;\mathrm{adult}\;\mathrm{males}\\ & +\mathrm{winner}\;\mathrm{age}\;\mathrm{of}\;\mathrm{oldest}\;\mathrm{male} \end{array}$$

Year, winner group ID, loser group ID, and unique pairings of groups (winner group ID + loser group ID) were included as random intercepts in each model to account for repeated measures of intergroup contests between the same groups and group dyads. Test statistics were obtained using the Anova function and confidence intervals using bootstrapping. We present the *P*-values, chi-squared values, parameter estimates (β) on the logit-scale, standard errors, and confidence intervals of each GLMM model, and compare *P*-values and direction of parameter estimates to the assumptions of pure self-assessment, and mutual assessment (Fig. 1). The collective self-assessment hypothesis would be supported if there was a positive and statistically significant relationship between RHP predictor variables and escalation for losing groups (β > 0; *P* < 0.05) and a weaker relationship for winning groups (β > 0; no significance threshold) (Fig. 1a, b). The collective mutual assessment hypothesis would be supported if these same tests indicated a significant positive relationship for losing groups (β > 0; *P* < 0.05) and a significant negative relationship for winning groups (β < 0; *P* < 0.05) (Fig. 1c, d).

## Results

**Fig. 3.**
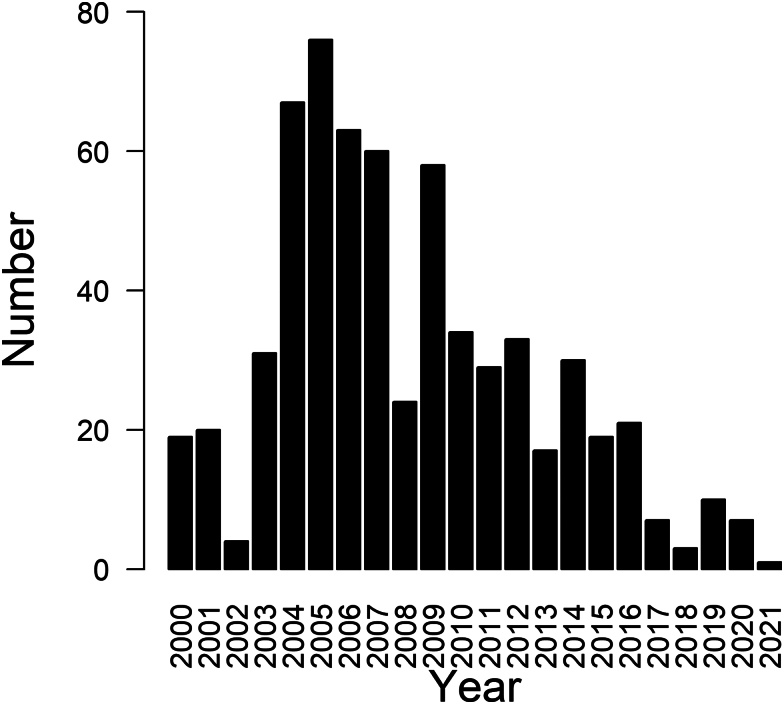
Number of observed intergroup contests in each year of the study period. (min = 1; max = 76; median = 22.50; mean = 28.77; *N* = 633).

Overall, of the 633 intergroup contests we observed, 280 were “non-physical” (43.7%), 230 were “physical” (35.9%), and 123 were undeterminable (20.4%). Across the 22 years, 2005 had the greatest number of observed intergroup contests (*N* = 76) whereas 2021 had the least (*N* = 1) (Fig. 3).

**Fig. 4.**
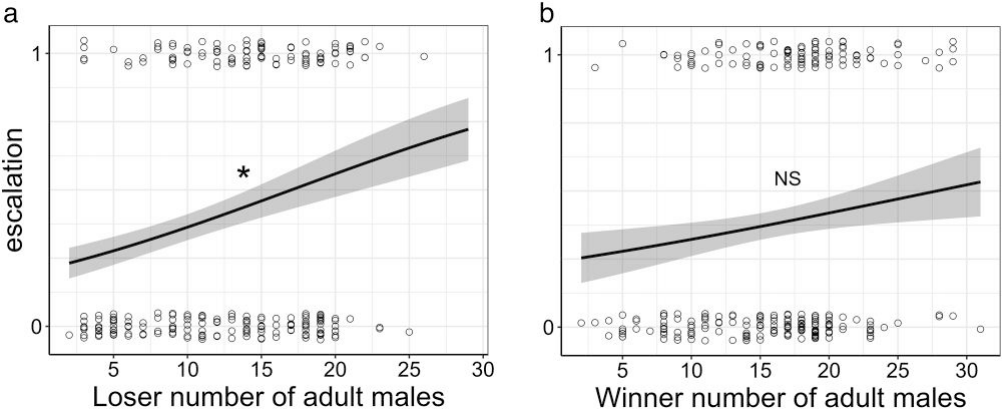
Support for collective self-assessment of number of adult males in banded mongooses. Probability of escalation plotted against (a) loser number of adult males (*P* = 0.005); (b) winner number of adult males (*P* = 0.18). Escalation was a binary metric where contests were either 0 = non-physical, or 1 = physical. The gray shaded area shows the standard error around the fitted line. An asterisk (*) denotes statistical significance while “NS” denotes lack of significance. Data (circles) represent individual contests and are randomly jittered on the y-axis. Data in plot includes both high and moderate confidence scores of our escalation metrics (*N* = 253).

Under the analysis where high and moderate confidence scores of our escalation metrics were combined (*N* = 253), our results supported self-assessment: there was a significant positive relationship between the probability of escalation and number of adult males for groups that lost fights (losers; β = 0.52, SE = 0.19, χ^2^ = 7.81, 95% CI [0.16, 0.94], *P* = 0.005), and a non-significant, positive relationship between these variables for groups that won fights (winners; β = 0.24, SE = 0.18, χ^2^ = 1.80, 95% CI [−0.12, 0.63], *P* = 0.18) (Fig. 4a, b; Table 1). Support remained for the self-assessment hypothesis when using only high confidence score escalation metrics (*N* = 229). Here, loser groups still showed a greater probability of escalation with larger numbers of adult males (losers; β = 0.57, SE = 0.21, χ^2^ = 7.51, 95% CI [0.19, 1.00], *P* = 0.006), with a non-significant, positive relationship between escalation and number of adult males for winners (winners; β = 0.33, SE = 0.20, χ^2^ = 2.77, 95% CI [−0.06, 0.80], *P* = 0.09; Table 1).

**Table 1.** Models predicting the probability of escalation for loser or winner RHP in intergroup contests.

Model escalation ∼ winner or loser RHP + (1 | year) + (1 | winner ID) + (1 | loser ID) + (1 | unique pairings)						
Dataset	RHP proxy	β	SE	*χ* ^2^	*P*	*CI (95%)*
**High and moderate confidence scores (*n* = 253)**	Number of adult males					
	Winner	0.24	0.18	1.80	0.18	−0.12, 0.63
	**Loser**	**0.52**	**0.19**	**7.81**	0.005*	**0.16, 0.94**
	Oldest male age (days)					
	Winner	0.11	0.15	0.51	0.47	−0.17, 0.47
	Loser	0.11	0.17	0.41	0.52	−0.25, 0.47
**High confidence scores only (*n* = 229)**	Number of adult males					
	Winner	0.33	0.20	2.77	0.09	−0.06, 0.80
	**Loser**	**0.57**	**0.21**	**7.51**	0.006*	**0.19, 1.00**
	Oldest male age (days)					
	Winner	0.18	0.17	1.11	0.29	−0.15, 0.55
	Loser	0.01	0.19	0.01	0.94	−0.36, 0.42

The estimate (β), standard error (SE), chi-squared value (χ^2^), *P*-value (P), and confidence interval (CI) of a model predicting the escalation of loser or winner RHP in intergroup contests.

Generalized linear mixed effects models with a binomial error structure and logit link function. Year, winner group ID, loser group ID, and unique pairings (winner group ID + loser group ID) were used as random intercepts (high and moderate confidence scores GLMM, *N* = 253 intergroup contests across 19 years and 12 winner group IDs, 20 loser group IDs, and 35 unique pairings) (high confidence scores only GLMM, *N* = 229 intergroup contests across 19 years and 11 winner group IDs, 19 loser group IDs, and 32 unique pairings). Significant terms are presented in **bold** and denoted by an asterisk (*). Note that parameter estimates are on the logit-scale.

There was no significant relationship between oldest male age for winners or losers and the probability of escalation in either confidence category, providing no statistical support for either model of assessment (Table 1).

## Discussion

We found support for the hypothesis that mongoose groups used collective self-assessment when deciding to escalate a contest into physical violence. Specifically, our results suggest that losing banded mongoose groups were more likely to escalate in contests when they had many adult males (a known proxy of RHP; (Green et al. 2022)), irrespective of the number of adult males in the winning group. By contrast, we found no support for either assessment model based on the oldest male age within the group. Despite assumptions that intergroup contests are determined through mutual assessment (eg, (Bennett and Stam 1996; Chan 2003; Radford 2003; Langlois and Langlois 2009; Stulp et al. 2012; Van Belle and Scarry 2015)) we found no support for this in our study.

At first sight our results are surprising. Why should banded mongooses assess only their own fighting force, and ignore potential valuable information about the outgroup? Research in dyadic contests has suggested that, while mutual assessment can minimize the costs of conflict by allowing the weaker party to back down earlier, acquiring information about an opponent may be constrained or entail prohibitively high costs (Mesterton-Gibbons and Heap 2014; Guillermo-Ferreira et al. 2015). For instance, assessing the strength of outgroups in the short time-window before conflict could be disproportionately costly, compared with own-group assessment which can be attained at relatively low cost over longer time periods such as through play-fighting and intragroup contests (Turner et al. 2020; Weller et al. 2020; Nolfo et al. 2021).

Our finding that the number of adult males in a banded mongoose group is associated with the probability of escalation accords with previous evidence of the importance of adult males for intergroup fighting success (Green et al. 2022). Males suffer greater mortality from conflict than females (Johnstone et al. 2020) and there is experimental evidence that in simulated encounters males were more likely to approach caged intruders (Cant et al. 2002). This suggests that males are the main participants in conflicts. It has also been suggested that males have evolved adaptations as a result, such as greater body mass and head size (Green et al. 2022), that may be important for fighting behavior (Gvoždík and Van Damme 2003; Wroe et al. 2005; Briffa et al. 2013; Chelliah and Sukumar 2013; Jennings and Gammell 2013; Vieira and Peixoto 2013) and determining contest success (Huyghe et al. 2005; Husak et al. 2006; McLean and Stuart-Fox 2015). Our findings are also consistent with the male warrior hypothesis, which proposes that male mammals possess specific adaptations for intergroup fighting and participate more in intergroup conflict than females, and thus have a pronounced effect on group RHP (Muñoz-Reyes et al. 2020; Smith et al. 2022). Therefore, the number of adult males is likely an important and informative RHP proxy which can be assessed when making decisions during intergroup contests in banded mongooses.

Having a numerically superior group appears to play such an important role in intergroup fighting because several attackers can completely overwhelm a lone opponent. In banded mongooses most fatalities seem to occur when individuals become isolated and overwhelmed by groups of attackers (*personal observations*; eg, Video 1). Outnumbering one's opponent presents many advantages such as the ability to attack simultaneously from multiple angles, to pin down an opponent while others deliver bites or blows, or to take turns in energetically intense fighting (Wilson et al. 2002; Adams and Mesterton-Gibbons 2003; Nunn and Deaner 2004; Baglione et al. 2010; Rusch and Gavrilets 2020). The importance of a bigger group for contest success is also emphasized in other animals such as meerkats (Dyble et al. 2019), primates (Majolo et al. 2020), lions (McComb et al. 1994; Mosser and Packer 2009), wolves (Cassidy et al. 2015), and ants (Batchelor and Briffa 2010). As discussed above, assessing own-group number of adult males may be a low-cost, more accurate way to use this information in competitive decision-making.

Despite the importance of oldest male age for banded mongoose group RHP (Green et al. 2022), the age of the oldest male in the group was not associated with escalation and did not show support for either assessment strategy. While age may play a role during intergroup contests and assessment in general (Fawcett and Johnstone 2010; Briffa and Lane 2017; Green et al. 2022), number of adult males may be a faster, simpler, or more reliable cue on which to base escalation decisions in intergroup contests. Assessing several attributes for more than one individual may be a cognitively demanding process (Johnson and Fowler 2013; Tecwyn et al. 2017; Budaev et al. 2019). Age in general may also be less conspicuous (Elwood and Arnott 2012; Fawcett and Mowles 2013; Nieder 2020), scalable or countable (Bonanni et al. 2011; Akre and Johnsen 2014; Nieder 2020) making estimates of age more prone to error. Additionally, in banded mongooses, the age of the oldest male was a weaker predictor of contest outcomes compared to number of adult males (Green et al. 2022), which is consistent with its weakness in association with contest escalation here.

Despite finding support for self-assessment in this analysis, intergroup conflict in banded mongooses does not fit classical assumptions of pure self-assessment models. The original self-assessment models assume no physical contact and that the costs of conflict are only a gradual escalation of display intensity over time (Bishop and Cannings 1978). In banded mongooses, frequent physical aggression and severe costs (eg, injuries, deaths) violate these assumptions (Cant et al. 2013; Thompson et al. 2017). This divergence mirrors findings in the dyadic contest literature, where violations of classical assumptions are widespread. For instance, Pinto et al. (2019) report that in 34 of 36 species in which self-assessment was supported, assumptions such as no physical contact and single-phase (display ritual) contests were violated. Many dyadic contest studies use a range of different proxies for cost, such as contest duration (Hsu et al. 2008; Briffa 2014), latency to approach and distance traveled (eg, (Beeching 1992; Wilson et al. 2011)), action rate (eg, (Hack 1997; Pratt et al. 2003; Jennings et al. 2012)), and escalation (Yasuda et al. 2012; McGinley et al. 2015; Green and Patek 2018). Subsequently, and mirroring calls in dyadic research (Pinto et al. 2019), we advocate for further theoretical development that builds additional realism into the study of assessment strategies (Kokko 2013; Mesterton-Gibbons and Heap 2014; Chapin et al. 2019).

Moving forward, key differences between individual and group contests may demand a more dedicated intergroup-specific theoretical framework. For example, within a population, assessment strategies may vary both within and between groups and may be context dependent. Chapin et al., (2019) discuss this variation with reference to individuals (dyadic contests) but a similar approach could be extended to groups. Also, not only do intergroup conflicts involve collective decision-making (Sankey et al. 2022) and variation in leadership dynamics (Hunt et al. 2024), but it is also possible that contests between groups could escalate into violence through mechanisms other than assessment-based decisions. For example, collective decisions to escalate into conflict may be dependent on the presence of a key individual (with an inherently risk-prone personality) already committed to escalation (Glowacki and McDermott 2022). No collective assessment is necessary, just a propensity for the group to follow those individuals which commit themselves to conflict initiation (the incentive for followers to join is thought to increase because key individuals pay a larger share of the startup costs—(Gavrilets and Fortunato 2014; De Dreu et al. 2016)). As part of our investigation here we modeled the dynamics of this alternative hypothesis but found continued support for collective self-assessment in banded mongooses (see Supplemental Material). In future work, expanding and formalizing intergroup-specific contest models will help determine the generality of various intergroup assessment (or non-assessment-based) strategies across taxa.

## Supplementary Material

araf133_Supplementary_Data

## Data Availability

Analyses reported in this article can be reproduced using the data provided by (Rayner et al. 2025).
