# Supplementary material for: Collective self-assessment in banded mongoose intergroup contests
Source: Behav Ecol. 2025 Nov 21;37(1):araf133. doi: 10.1093/beheco/araf133 (PMC12848321; doi:10.1093/beheco/araf133)
Supplement: araf133_Supplementary_Data [file araf133_Supplementary_Data.docx]

**Supplemental Material.**

Testing a Non-Assessment-Based Hypothesis: The “Key Individuals” Model.

An alternative hypothesis to assessment (only relevant in groups) is the “key individuals” hypothesis. Here, collective decisions to escalate into conflict may be dependent on the presence of a key individual (with an inherently risk-prone personality) already committed to escalation (Glowacki & McDermott, 2022). No global assessment is necessary, just a propensity for the group to follow those individuals which commit themselves to conflict initiation. It has been reasoned that followers have more incentive to join when key individuals pay a larger share of the startup costs (De Dreu et al., 2016; Gavrilets & Fortunato, 2014). Assuming that larger groups would be more likely to contain at least one key individual, this hypothesis has potential to fit our data, as we observed that larger groups are more aggressive. It is important that we distinguish between this alternative hypothesis and assessment-based explanations. We now detail a simple simulation model aimed at generating predictions to tease apart assessment-based and ‘key individuals’ hypotheses.

In the self-assessment model, for each of N=1000 intergroup contests, two groups are formed with group sizes randomly drawn (with replacement) from group sizes in our dataset of intergroup encounters (N=904). Each group estimate their own group size (by a combination of individual assessments – mechanism not specified) with some margin of error, such that estimated group size is drawn from a Gaussian distribution with mean = group size, and S.D. = estimation bias parameter (λ). This bias parameter can vary, and is tested at λ=3, 7, and 15 in both self-assessment and mutual assessment simulations. If the group collectively estimates that they are at or above a threshold *φ* = 23 (the median group size in our encounters dataset)*,* they will choose to escalate to a physical fight. If both groups escalate, the winner is the larger group (or a randomly selected group when group sizes are equal). If only one group is above the threshold, they chase the other group away; no conflict occurs, but the chaser is said to have “won” the contest (Table 2). The conflict outcomes, therefore, are chases (with a winner group and a loser group), and fights (with a winner group and a loser group). This matches how we have treated and classified intergroup interactions in our analyses of the empirical data. When both teams estimate themselves to be below the threshold *φ*, no conflict occurs and there is no winner or loser.

**Table 2**. Whether each group escalates, the outcome and the winner.

| Group 1 escalate? | Group 2 escalate? | Intensity | Winner |
| --- | --- | --- | --- |
| T | F | Non-physical | Group 1 |
| F | T | Non-physical | Group 2 |
| T | T | Physical | Larger group * |
| F | F | NA | NA |

*or randomly selected winner if groups are the same size

The mutual-assessment model has the same number of contests as above, and group formation is the same as above. Here, each group size is estimated, with estimation bias of λ= 3, 7 and 15 as above. Both teams use the difference between their estimates to decide whether to escalate, with rule: escalate if they estimate their group to be larger. “Physical” conflict occurs when both groups estimate themselves to be larger. Fight winners and losers are determined the same way as the self-assessment model (Table 2).

In the ‘key individuals’ model, the number of contests and group formation remains the same as previous models. Here, however, each individual in the group has a probability p=0.05 of being a key individual (p=0.05 creates a ratio of non-physical to physical encounters which closely matches our empirical data (Fig. S1)). If the group contains at least one key individual, the group will escalate. Winner/loser determination is consistent with self- and mutual-assessment models (Table 2).

The self-assessment model is the most consistent with our data (Fig. S2). The ‘key individuals’ model fails to predict losing groups having a steeper relationship between loser escalation and group size than winner escalation and group size; losers have an inverse U-shaped curve, which reflects the fact that large group sizes are usually only losers if they do not escalate (Fig. S2E, F). The self-assessment model, on the other hand, recreates the qualitative pattern from our empirical data (Fig. S2A, B). Reassuringly, the mutual assessment model follows the expectations from the dyadic contest assessment literature – that of winners having a negative slope between the cost proxy (escalation) and RHP (group size) (Fig. S2C, D). One inconsistency between the data and the self-assessment model is that when the estimation bias parameter λ is extremely large (λ=15), the slope of the relationship between winner group size and intensity can exceed the loser’s slope the self-assessment model (Fig. S3). This could indicate that accurate RHP estimation is a necessary component for a self-assessment conclusion. A fuller exploration of this inconsistency could certainly prove fruitful, yet a more formal expansion of the model is outside of the scope for this paper.

The model served two purposes. Firstly, it increased our confidence that banded mongooses use self-assessment strategies. Second, this model can now be built-upon to test contest strategies more explicitly in intergroup conflict. Key differences between individual and group contests demand a more dedicated theoretical framework. Group conflicts involve collective decision-making (Sankey et al., 2022), variation in leadership dynamics (Hunt et al., 2024), and potentially complex estimation of self (and/or relative) strength, all of which shape escalation patterns in ways that dyadic models cannot fully capture. Building and then testing, purpose built, intergroup-specific models will help determine the generality of various intergroup assessment (or non-assessment-based) strategies across taxa.

**
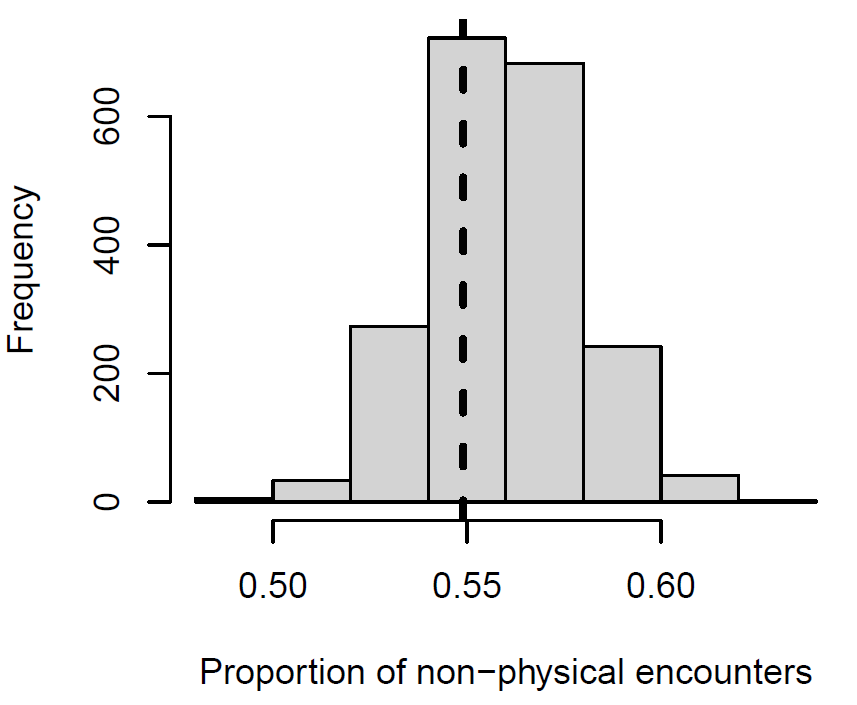
**

**Figure S1.** Under a “key individuals” hypothesis, p=0.05 was chosen as the probability of each individual being a key individual. This probability, when run through the “key individuals” version of the model, translates into a similar proportion of non-physical encounters, to that which we saw in the empirical data (black segmented line). Histograms show the frequency of non-physical encounter proportions from 1000 iterations of N=1000 intergroup encounters.


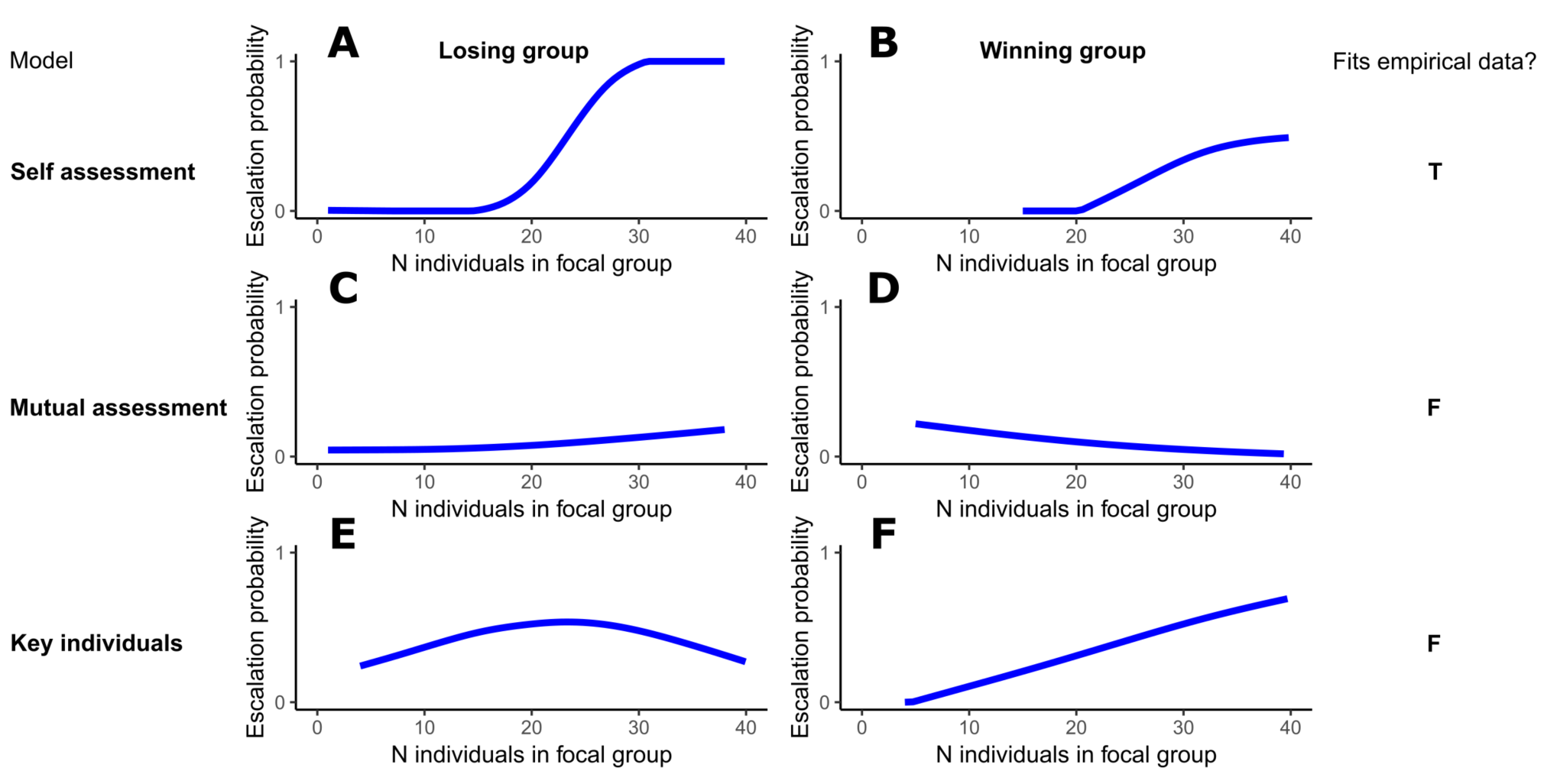


**Figure S2. Model results.** See *in text* for model mechanism, analysis of results and conclusions. Line plotted fitted with generalized additive models (GAM) in ggplot2 (REF).


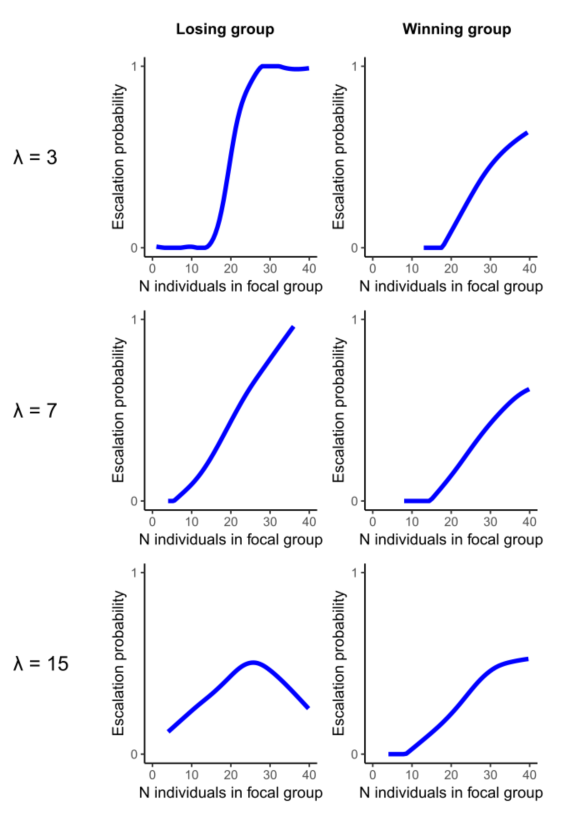


**Figure S3.** Escalation over group size for different estimation bias parameters (rows), and for winning and losing groups (columns). When estimation of group size is extremely noisy (bottom row) loser slopes (left column) decrease past a certain point (roughly 25 individuals which is just over the median group size in the mongoose encounters dataset; median = 23), and fail to convincingly match our data which suggests greater slopes for loser escalation than winner escalation.

**Video S1.** When one group greatly outnumbers another, individuals become isolated and overwhelmed.

**References**

De Dreu, C. K. W., Gross, J., Méder, Z., Giffin, M., Prochazkova, E., Krikeb, J., & Columbus, S. (2016). In-group defense, out-group aggression, and coordination failures in intergroup conflict. *Proceedings of the National Academy of Sciences of the United States of America*, *113*(38), 10524–10529. https://doi.org/10.1073/pnas.1605115113

Gavrilets, S., & Fortunato, L. (2014). A solution to the collective action problem in between-group conflict with within-group inequality. *Nature Communications*, *5*(1), 1–11. https://doi.org/10.1038/ncomms4526

Glowacki, L., & McDermott, R. (2022). Key individuals catalyse intergroup violence. In *Philosophical Transactions of the Royal Society B: Biological Sciences* (Vol. 377, Issue 1851). The Royal Society. https://doi.org/10.1098/rstb.2021.0141

Hunt, K. L., Patel, M., Croft, D. P., Franks, D. W., Green, P. A., Thompson, F. J., Johnstone, R. A., Cant, M. A., & Sankey, D. W. E. (2024). The evolution of democratic peace in animal societies. *Nature Communications 2024 15:1*, *15*(1), 1–8. https://doi.org/10.1038/s41467-024-50621-5

Sankey, D. W. E., Hunt, K. L., Croft, D. P., Franks, D. W., Green, P. A., Thompson, F. J., Johnstone, R. A., & Cant, M. A. (2022). Leaders of war: Modelling the evolution of conflict among heterogeneous groups. *Philosophical Transactions of the Royal Society B: Biological Sciences*, *377*(1851). https://doi.org/10.1098/rstb.2021.0140
